# Correction of Anterior Open Bite Using Temporary Anchorage Devices: A Systematic Review and Meta-Analysis

**DOI:** 10.3390/jcm14144958

**Published:** 2025-07-13

**Authors:** Patricia Burgos-Lancero, Marta Ibor-Miguel, Laura Marqués-Martínez, Paula Boo-Gordillo, Esther García-Miralles, Clara Guinot-Barona

**Affiliations:** Department of Dentistry, Faculty of Medicine and Health Sciences, Catholic University of Valencia San Vicente Mártir, 46001 Valencia, Spain; pburgosl@mail.ucv.es (P.B.-L.); marta.ibor@ucv.es (M.I.-M.); paula.boo@ucv.es (P.B.-G.); esther.garcia@ucv.es (E.G.-M.); clara.guinot@ucv.es (C.G.-B.)

**Keywords:** anterior open bite, temporary anchorage devices, molar intrusion, skeletal anchorage, vertical malocclusion, systematic review, meta-analysis

## Abstract

**Background/Objectives:** Anterior open bite (AOB) is a complex malocclusion characterized by the lack of vertical overlap between the upper and lower teeth during maximum intercuspation. It often results in functional impairments and aesthetic concerns. Traditional treatments for adult patients, including orthognathic surgery, are effective but invasive. Temporary anchorage devices (TADs) have emerged as a minimally invasive alternative. The aim of this systematic review and meta-analysis was to evaluate the effectiveness of TADs for molar intrusion in the correction of AOB. **Methods:** A systematic review was conducted according to the PRISMA 2020 guidelines. An electronic search was performed in PubMed and Scopus until March 2025. The inclusion criteria comprised clinical studies in humans published in English or Spanish in the last 10 years. The risk of bias was assessed using RoB 2, ROBINS-I, and the Joanna Briggs Institute tools. A random-effects meta-analysis was carried out to estimate pooled intrusion values, and heterogeneity was evaluated using Cochran’s Q test and the I^2^ statistic. **Results:** Twelve studies were included. Molar intrusion using TADs achieved significant overbite improvements, with a pooled mean intrusion of 1.70 mm (95% CI: 0.53–2.87 mm). The heterogeneity among studies was high (I^2^ = 88.5%). Despite variability in force magnitude and TAD type, lighter forces were generally associated with similar outcomes and fewer adverse effects. **Conclusions:** TADs offer a predictable and less invasive alternative to orthognathic surgery for AOB correction. When appropriately indicated and biomechanically managed, they provide effective vertical control and short- to medium-term stability in adult patients.

## 1. Introduction

Anterior open bite (AOB) is characterized by the absence of vertical overlap or contact between the upper and lower teeth during maximum intercuspation [1]. This condition may be localized in the anterior region, affecting incisor alignment, or in the posterior region, involving the molars [2].

AOB is a multifactorial malocclusion resulting from the interaction of genetic, skeletal, dental, environmental, and functional factors [3]. It affects not only facial aesthetics but also essential functions such as speech, swallowing, and mastication, compromising quality of life and social interaction. Therefore, understanding its multifactorial nature is essential for a comprehensive diagnosis and treatment approach that addresses both the predisposing and perpetuating factors of this condition [4].

Open bite is one of the most challenging malocclusions in orthodontics due to the difficulty of achieving long-term stability [5]. As a result, skeletal anchorage devices such as miniplates and miniscrews are currently widely used in orthodontics for their ability to provide reliable anchorage throughout treatment. These devices allow for the correction of skeletal AOB by facilitating molar intrusion, which is more effective and less invasive than anterior tooth extrusion, and promotes a counterclockwise mandibular rotation that contributes to bite closure [3].

Although orthognathic surgery has traditionally been the treatment of choice for skeletal anterior open bite [6], the use of miniplates and miniscrews has proven to be a less invasive alternative with lower risk and faster recovery. The advent of skeletal anchorage and the use of cone beam computed tomography (CBCT) have enabled greater diagnostic precision and more predictable treatment planning, reducing the need for orthognathic surgery [7]. In addition, the use of temporary anchorage devices (TADs) has improved treatment stability, particularly in cases involving skeletal dysplasia that previously required surgical correction [5].

TADs are tools used in orthodontics to enhance anchorage control, enabling more predictable and controlled tooth movements [6,8]. The use of TADs has broadened the scope of conservative treatment options, enabling effective intrusion of the buccal segments in the maxilla—and occasionally in the mandible. These devices are temporarily fixed to the bone and removed once their function has been fulfilled [9], facilitating orthodontic treatment without relying exclusively on patient compliance [10].

Miniscrews are one of the most commonly used forms of TADs, due to their simplicity and ease of use. They are generally made of biocompatible materials such as titanium, which is corrosion-resistant and exhibits excellent osseointegration, making it ideal for use in the human body [8].

TADs offer great versatility and represent a less invasive alternative to orthognathic surgery. Their use in orthodontics allows for more efficient and predictable tooth movements, such as molar intrusion or tooth movement without affecting adjacent structures. Moreover, their rapid placement and removal once the treatment objective is achieved make them highly popular in modern orthodontics. The use of TADs has also reduced treatment costs and duration, with their success largely dependent on the correct selection of the insertion site and placement technique.

The objective of the present study was to evaluate the effectiveness of temporary anchorage devices (TADs) in molar intrusion as a treatment for correcting anterior open bite in adults (Figure 1).

## 2. Materials and Methods

### 2.1. Protocol and Registration

A systematic review of the literature was conducted in accordance with the PRISMA 2020 guidelines (Preferred Reporting Items for Systematic Reviews and Meta-Analyses).

### 2.2. Information Sources

Articles evaluating the effectiveness, stability, and potential side effects of this orthodontic treatment modality were collected and assessed to provide an updated and critical overview of the management of this malocclusion using skeletal anchorage.

An electronic search was conducted in the Medline (PubMed), Scopus, and Embase databases to identify potentially relevant studies. In specific cases, the authors of the studies were contacted by email to request further information. A manual search of the reference lists of included studies was also performed to identify any additional articles not found in the databases. The last search update was conducted in March 2025.

The following PICO question was formulated for this systematic review (Patients, Intervention, Comparison, Outcome):

- P (Patients): Patients with anterior open bite
- I (Intervention): Molar intrusion using temporary anchorage devices
- C (Comparison): Other treatment modalities for correcting open bite, such as orthognathic surgery
- O (Outcomes): Molar intrusion with TADs as an effective and less invasive technique, associated with lower risk and faster recovery.

### 2.3. Search Strategy

A combination of Medical Subject Headings (MeSH) and free-text terms was used to construct the search strategy. The Boolean operators “AND” and “OR” were employed to link related terms and include synonymous expressions relevant to the systematic review topic. The complete list of terms used is presented in Table 1.

By applying these operators, the final search string (#6) was as follows:

((humans) AND ((((open bite) OR (dolichofacial)) OR (vertical malocclusion)) OR (vertical dysplasia)) AND (((orthodontic anchorage procedures) OR (bone screws))) AND (tooth intrusion)) AND (((efficiency) OR (effectiveness)) OR (amount intrusion))).

After executing the search, all retrieved articles were imported into Zotero to identify and eliminate duplicates.

### 2.4. Eligibility Criteria

The eligibility criteria for study inclusion were defined with the aim of evaluating the effectiveness of temporary anchorage devices (TADs) in molar intrusion as a treatment for anterior open bite correction.

Inclusion criteria:

- Articles published within the last 10 years
- Articles written in English or Spanish
- Randomized controlled trials (RCTs), cohort studies, case-control studies, and case series
- Human studies

Exclusion criteria:

- Systematic reviews and meta-analyses

### 2.5. Data Extraction

The search strategy was carefully designed, taking into account prior research in the field and the most frequently cited descriptors. To ensure accuracy, duplicate references were identified by cross-referencing and subsequently imported into Mendeley reference management software 2.x.

Two independent reviewers (P-BL and L-MM) assessed the titles and abstracts of all identified articles. In cases of disagreement, a third reviewer (E-GM) was consulted to reach a consensus. When abstracts lacked sufficient information, full-text articles were reviewed to determine eligibility.

Articles that met the predefined criteria were selected for inclusion in the review. Several parameters were established to assess the characteristics, methodology, and outcomes of each study. Authorship and year of publication were used to differentiate the studies. Data on failure and success rates, as well as clinical variables, were collected. The main findings and conclusions of each included study were integrated into the outcome variables for a comprehensive analysis.

### 2.6. Study Selection and Variables

The titles of all articles retrieved from PubMed and Scopus were systematically reviewed. A total of 31 articles were initially selected for inclusion in this systematic review. The Oxford Centre for Evidence-Based Medicine (OCEBM) levels of evidence, which classify scientific studies according to the strength and reliability of the evidence they provide in clinical practice (Figure 2), were subsequently applied.

As this was a systematic review, randomized controlled trials (RCTs) were prioritized. However, only three RCTs were found. Therefore, the inclusion was extended to cohort studies (six articles) and case-control studies (13 articles), which fall under Level V of the evidence hierarchy. Abstracts of all identified articles were read; when the abstract did not provide sufficient information to determine eligibility, the full text was reviewed. Based on this process, five articles were excluded.

All ineligible articles were excluded with clear justification.

The following variables were extracted from each included study: author, year, and journal, study design, sample size, type of technique used to correct the open bite, treatment duration, amount of molar intrusion achieved after treatment, increase in overbite, use of temporary anchorage devices (TADs), and type of TAD employed.

### 2.7. Risk of Bias

To assess the risk of bias in randomized controlled trials (RCTs), the RoB 2 tool developed by the Cochrane Collaboration was used. This tool evaluates five key domains:

1. Bias arising from the randomization process
2. Bias due to deviations from intended interventions
3. Bias due to missing outcome data
4. Bias in measurement of the outcome
5. Bias in selection of the reported result

For observational clinical studies, the ROBINS-I tool (Risk Of Bias In Non-randomized Studies—of Interventions), also developed by Cochrane, was applied. This tool evaluates seven domains:

1. Confounding
2. Selection of participants
3. Classification of interventions
4. Deviations from intended interventions
5. Missing data
6. Measurement of outcomes
7. Selection of the reported result

Finally, for case reports, the JBI (Joanna Briggs Institute) Critical Appraisal Tool was used. This tool offers a flexible and specific evaluation based on the type of study design. Each checklist contains closed questions (yes, no, unclear, not applicable) regarding aspects such as the clarity of the research question, inclusion criteria, measurement validity, confounding factors, statistical analysis, and appropriateness of follow-up. It does not provide an overall score but enables a structured assessment of overall quality.

### 2.8. Data Synthesis and Statistical Analysis

Meta-analyses were conducted based on clinical parameters including the ratio of micro-implants per tooth, the magnitude of the applied force, and the duration of treatment.

Study heterogeneity was assessed using Cochran’s Q test and the I^2^ index, with significant heterogeneity defined as *p* < 0.05 in the Q test and high heterogeneity indicated by an I^2^ value greater than 50% [6]. Due to substantial heterogeneity, a random-effects model was applied for the meta-analysis [3].

Publication bias was evaluated through visual inspection of funnel plots and by applying Egger’s test [11].

## 3. Results

### 3.1. Study Selection Characteristics

The electronic search yielded a total of 60 results: 41 from PubMed and 19 from Scopus. After removing duplicates, 51 studies remained. Following the screening of titles, abstracts, and full-texts, 19 studies were excluded for not meeting the inclusion criteria. As a result, 12 studies were included in the final systematic review.

The PRISMA flow diagram (Figure 3) provides a visual summary of the study selection process.

### 3.2. Results of Individual Studies

All studies included in this review shared a common therapeutic goal: the use of molar intrusion to correct anterior open bite with temporary anchorage devices (TADs) in patients with no or minimal growth. The outcomes demonstrated statistically significant and clinically stable results in the short to medium term.

The included studies were observational in design with interventional components. They generally followed a similar structural pattern, including a comprehensive summary, a clear and concise objective, a detailed methodology, and a statement of the most relevant conclusions.

After the selection of studies and their critical appraisal, the most relevant data analyzed in each study were extracted and are summarized in the following table (Table 2).

### 3.3. Quality Assessment

The table presents the risk of bias assessment of the eight observational clinical studies on molar intrusion for open bite correction using temporary anchorage devices (TADs). It includes seven domains, each with specific signaling questions to which responses were given as “Yes”, “No”, or “Partially” (Table 3).

On the other hand, to assess the risk of bias of the randomized clinical trials, the RoB 2 tool [23] was used. This tool evaluates five key domains, as illustrated in the following table (Table 4).

Table 4 shows the application of the RoB 2 tool to the randomized clinical trials included in this review [13,14]. Both studies used computer-generated randomization and sealed opaque envelopes, ensuring adequate allocation concealment, and were therefore judged at low risk for the randomization process.

Although participants and outcome assessors were blinded, operators were not, raising some concerns in the domain of deviations from intended interventions. However, there were no protocol deviations, and analyses followed the intention-to-treat principle.

Regarding outcome measurement, both studies employed valid and objective methods, such as CBCT imaging and digital models, and evaluator blinding was documented. There was no evidence suggesting that knowledge of group assignment influenced the measurements; therefore, this domain was rated as having a low risk of bias.

Finally, for the selection of the reported result, both trials reported the outcomes that were pre-specified (molar intrusion [14] and root resorption [13], with no indication of selective reporting, and were thus also judged to be low-risk. Overall, both studies were assessed as having a low risk of bias, with some minor concerns due to the lack of operator blinding during the clinical intervention.

Finally, the JBI critical appraisal tool [24] was used for the case report, which consists of eleven questions answered as “Yes”, “No”, “Unclear”, or “Not applicable”, as shown in Table 5.

This table presents the appraisal of the study by Kaku et al., which demonstrates good methodological quality despite its inherent limitations as a case report, particularly in the description of diagnosis, intervention, and outcomes.

According to the GRADE framework, the overall certainty of evidence for molar intrusion using TADs in anterior open bite cases was considered moderate. This assessment accounts for:

- A predominance of observational studies with moderate risk of bias, as evaluated through ROBINS-I and JBI tools.
- Limited consistency in reported outcomes, with intrusion values ranging from 1.0 mm to 3.1 mm.
- Sample size variability and occasional absence of confidence intervals, affecting precision.

Despite these limitations, the presence of two randomized controlled trials and multiple cohort studies with well-documented protocols support a moderate level of confidence in the results.

### 3.4. Quantitative Synthesis

In addition to the qualitative synthesis, a complementary single-mean meta-analysis was performed, which pooled data from five clinical groups extracted from four of the included studies. All groups used temporary anchorage devices (TADs) to achieve molar or premolar intrusion in adult patients.

The following data were collected for each group: number of subjects (*n*), mean amount of intrusion (in mm), standard deviation (SD), and force applied (in grams).

A random-effects model (DerSimonian-Laird) [25] was used to estimate the combined mean intrusion. This model is appropriate when true effects are expected to vary across studies due to methodological or clinical differences, as was the case here.

The following analyses were conducted:

1. Pooled mean calculation (Figure 4):

Weighted means were calculated using the inverse variance method under a random-effects model.

2. 95% confidence interval (CI) estimation (Figure 5):

A CI was calculated for the pooled mean to assess the precision of the overall effect.

3. Heterogeneity analysis:
  - Cochran’s Q test: Quantified observed variability among studies.
  - I^2^ statistic: Represented the percentage of variability attributable to true heterogeneity rather than chance. An I^2^ > 75% indicates high heterogeneity.

In accordance with the PRISMA guidelines, the results of each individual study were presented along with summary data for each intervention group, including effect estimates and their corresponding confidence intervals [25]. A forest plot was used to visualize the individual and combined effects across studies.

As previously noted, five clinical groups from four studies were included. All evaluated molar or premolar intrusion using TADs in adult patients. The forces applied ranged from 110 g to 400 g, and the number of subjects per group ranged from 10 to 19.

The pooled mean intrusion, estimated using a random-effects model, was 1.70 mm (95% CI: 0.53–2.87 mm), which is considered clinically relevant. The heterogeneity analysis yielded Q = 34.72 and I^2^ = 88.5%, indicating high variability among the studies.

A simple linear regression model was applied to explore the relationship between the applied force (in grams) and the amount of intrusion (in millimeters). Although a slightly positive slope was observed—suggesting that greater force may lead to greater intrusion—the dispersion of data points and the distribution of values indicated that the relationship is neither linear nor directly proportional. This supports the physiological hypothesis that excessive forces may lead to adverse effects, such as periodontal ligament necrosis, which hinders efficient continuous tooth movement.

The results suggest that TADs enable significant molar and premolar intrusion in adult patients. However, the high I^2^ value reflects considerable differences in clinical protocols, including the magnitude of applied force.

A key finding is that increasing the applied force does not necessarily result in greater intrusion. For example, the group receiving 300 g of force (Al-Fahali et al.) [12] showed greater mean intrusion than the group receiving 400 g (Akl et al.) [13,14]. This phenomenon may be explained by the biological effects of overloading: excessive force can compromise the periodontal ligament’s vascular supply, leading to hyaline necrosis. This damage requires a repair process before tooth movement can resume, thereby delaying or reducing the expected intrusion efficiency.

Therefore, using moderate forces—sufficient to stimulate bone remodeling without causing necrosis—maximizes efficiency while minimizing biological complications.

## 4. Discussion

Anterior open bite (AOB) is one of the most challenging malocclusions in orthodontics, due to both its multifactorial etiology and the complexity of achieving long-term correction and stability. This condition can significantly impact facial aesthetics, masticatory function, speech, and the patient’s overall quality of life. Conventional treatment in adults—particularly in moderate to severe cases—has traditionally involved orthognathic surgery. However, this option is invasive, costly, and not always accepted by patients.

In recent decades, the development of temporary anchorage devices (TADs) has revolutionized orthodontic biomechanics, enabling controlled tooth movements that were previously difficult to achieve. In particular, upper molar intrusion using TADs has emerged as an effective alternative for treating AOB in adult patients. This technique not only avoids surgery, but also allows controlled vertical correction, improves overbite, and often induces favorable mandibular rotation. The reviewed studies consistently support these benefits, although clinical effectiveness, magnitude of changes, and long-term stability vary depending on study design, biomechanical protocol, and type of TAD used.

For example, a study by Lee et al. (2024) [15] is one of the few comparing TAD-based intrusion with orthognathic surgery, showing similar functional results regarding bite force and occlusal contact area after two years of follow-up. Their protocol—using interradicular miniscrews between upper premolars and molars, light forces of 20–30 g per tooth, and controlled mechanics—resulted in 1.7 mm of molar intrusion and a 4 mm overbite increase. This approach proved to be predictable, minimally invasive, and stable over time, making it the most recommendable clinical protocol according to the reviewed evidence.

Similarly, Ogura et al. (2024) [8] reported 1.6 mm of intrusion using miniscrews in the posterior maxilla, resulting in a 4.1 mm overbite increase with complete stability at one year. Their protocol involved 200 g of force per side for seven months and included 3D analysis, providing robust control over tooth movement and reinforcing the utility of this technique in mild to moderate AOB cases. However, this protocol requires greater technological resources compared to others, and although the results are similar, the level of evidence of this study is lower than that of the previously described cohort study by Lee et al.

Studies by Marzouk et al. (2015 and 2016) [16,17] used zygomatic miniplates and higher forces (450 g), achieving intrusions over 3 mm and overbite increases close to 7 mm, along with counterclockwise mandibular rotation and significant aesthetic improvement. Despite their high efficacy, particularly in severe AOB cases, relapse rates of 10–13% were reported after four years, highlighting the importance of a strict retention protocol. Additionally, their more aggressive surgical approach should be considered. While informative, the 2015 study had a high risk of bias, and neither study offers high-level evidence: the 2016 article, though longitudinal, lacked a comparison group (not a true cohort study), and the 2015 article was a non-randomized clinical trial.

Peres et al. (2023) [18] provided a clear quantitative analysis, directly correlating 2.67 mm of intrusion with 3.6 mm of bite closure. Their predictive model estimated that each millimeter of intrusion yields a 0.86 mm increase in overbite. The study included an age-matched untreated control group (n = 12) for comparison, providing a moderate level of evidence. Their clinical protocol—using palatal miniscrews and a transpalatal arch—offered an efficient, side-effect-free approach.

The RCT by Akl et al. (2020) [14], one of the highest-quality studies among those included in the analysis, showed that both 200 g and 400 g of force achieved similar results (≈2.4 mm intrusion, ≈2.7 mm overbite increase), with lower biological stress in the lighter-force group. This supports the use of gentler protocols that maintain efficacy while improving safety.

Conversely, Al-Falahi et al. (2018) [13] and Akl et al. (2021) [14] focused on treatment side effects—mainly root resorption. Although statistically significant, the resorption observed was clinically minor and not a threat to treatment success under well-controlled protocols. These studies were included for reference but did not aim to correct open bite specifically. Moreover, Al-Falahi’s study had a high risk of bias and was a non-randomized interventional study—like [17]—so it does not qualify as a case-control or cohort study despite belonging to the same evidence level.

The study by Akan et al. (2020) [19], though showing high effectiveness (2.32 mm intrusion, 2.48 mm overbite increase), had greater methodological risk due to its retrospective design and lack of a control group. Despite this, it supports the use of zygomatic miniplates in open bite treatment.

In contrast, Lo-Cao et al. (2025) [20] compared two adolescent protocols: Invisalign aligners and the SIS system with TADs. While both closed the open bite, only the SIS group achieved true molar intrusion and acceptable occlusal quality. These findings question the effectiveness of aligners alone in such cases and highlight the utility of TADs. However, this study involved adolescents and had limited follow-up, so its relevance to adult treatment remains to be confirmed.

Finally, Guo et al. (2024) [21] demonstrated that adding vertical control via TADs to sagittal anchorage protocols significantly improved facial profile, achieving 2.25 mm of intrusion and mandibular counterclockwise rotation. This highlights the aesthetic and functional benefits of combining TADs with individualized vertical planning, though it also requires advanced technological resources.

A case report by Kaku et al. (2019) [22], despite its low evidence level, offered an interesting approach to managing open bite in a patient with temporomandibular disorder (TMD). The case was treated solely with orthodontics using miniscrews, avoiding surgery through precise biomechanical planning. Although multiple extractions were performed and incisor extrusion played a greater role than molar intrusion (the main focus of this review), the case illustrates the potential of well-designed conservative alternatives in complex scenarios.

Overall, the evidence supports the efficacy of TADs as a conservative and predictable alternative to orthognathic surgery—particularly when using light forces, selecting appropriate anchorage types (miniplates or miniscrews), and ensuring long-term retention protocols. The most robust protocol is that of Lee et al., followed by those of Lo-Cao, Ogura, and Guo, with the caveat that more adult data and longer follow-up are needed for Lo-Cao’s findings.

When considering both effectiveness and overall clinical efficacy, the most valuable articles in this review—aside from Lee et al., which is the most comprehensive—are those by Peres et al. [18] and Akl et al. [14]. Standardizing these protocols and selecting patients appropriately are essential to achieving stable and clinically meaningful outcomes.

Despite the encouraging outcomes, this systematic review presents several limitations that should be considered. First, there was considerable heterogeneity among studies in terms of clinical protocols, sample characteristics, types of TADs, force magnitude, and treatment duration. This variability, confirmed in the meta-analysis, limits direct comparability but provides a broader estimate of the intervention’s effectiveness.

Second, the methodological quality of many studies was moderate to low. The limited number of randomized controlled trials required the inclusion of observational designs, which reduced the overall strength of evidence.

Another key limitation is the lack of long-term follow-up, which is critical given the high relapse rate of anterior open bite. Without post-treatment data, long-term stability remains uncertain.

Moreover, although greater force does not translate proportionally into faster or greater intrusion, there is insufficient evidence to establish optimal force protocols. Future studies should standardize force application and correlate it with the degree of intrusion and possible adverse effects, such as root resorption.

Finally, the growing variety of TADs used for molar intrusion highlights the need to compare patient-reported comfort across different miniscrew types—an important factor that may influence adherence and overall clinical outcomes.

## 5. Conclusions

Following the development of this systematic review and the analysis of all included articles, the following conclusions can be drawn:

- Temporary anchorage devices (TADs) are an effective and safe tool for molar intrusion in the treatment of anterior open bite, particularly in adult patients.
- Comparison between different placement techniques shows that both interradicular miniscrews and zygomatic miniplates can be effective, provided they are applied using well-controlled mechanical protocols.
- Regarding clinical protocols, evidence supports individualized planning. Nevertheless, the most stable outcomes with fewer complications are associated with light forces, segmented arch mechanics, and TADs placed in biomechanically safe areas such as the palate or buccal interradicular region, following prior cephalometric evaluation.
- The meta-analysis revealed a mean molar intrusion of 1.7 mm, which is clinically significant. However, the high heterogeneity among studies highlights the need for further research to determine the achievable degree of intrusion and to define optimal force protocols, as excessive forces do not proportionally increase intrusion and may compromise outcomes.

## Figures and Tables

**Figure 1:**
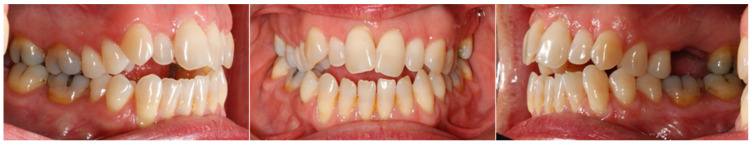
Intraoral images of an open bite clinical case.

**Figure 2:**
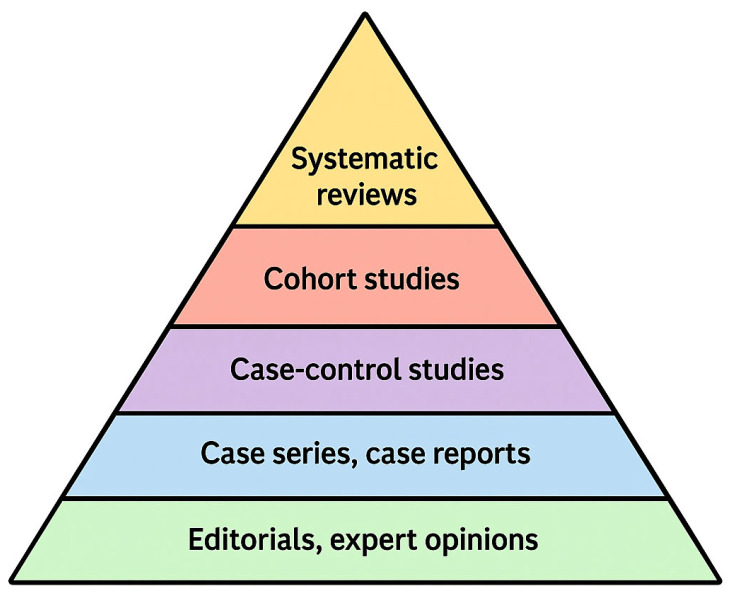
Pyramid of scientific evidence.

**Figure 3:**
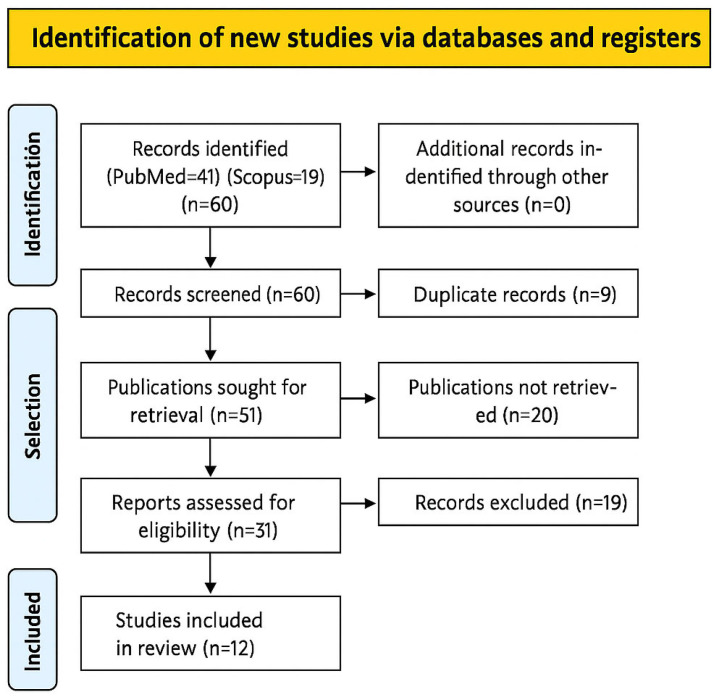
PRISMA flow diagram of study selection. The PRISMA flow diagram.

**Figure 4:**
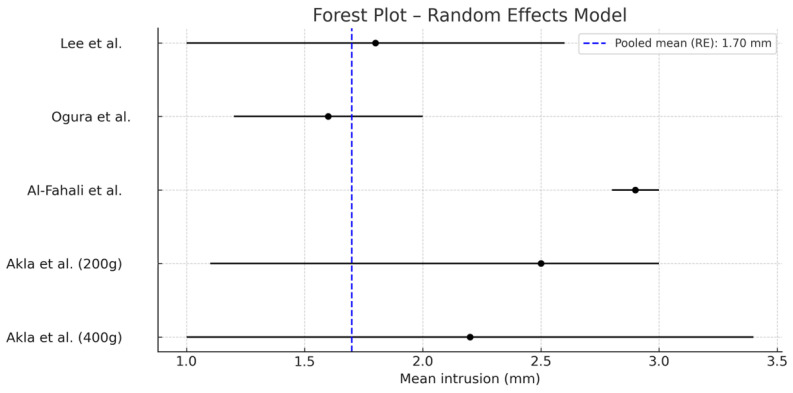
Forest plot showing a random-effects model for individual studies [8,12,14,15].

**Figure 5:**
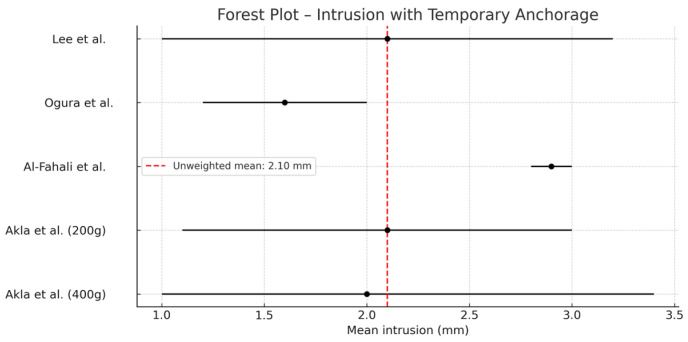
Forest plot showing effect sizes and 95% confidence intervals for each included study [8,12,14,15].

**Table 1:** Mesh and free-text terms used in the search strategy.

Patients	Intervention	Comparison	Outcomes
#1 Humans [MeSH]	#3 Orthodontic anchorage devices [MeSH] Skeletal anchorage Bone screws [MeSH] OR		#5 Efficiency Efectiveness Amount intrusion OR
#2 Open bite [MeSH] Dolichofacial Vertical malocclusion Vertical dysplasia OR			
	#4 Tooth intrusion [MeSH]		
#1AND #2AND#3AND#4AND #5 = #6			

**Table 2:** Data extracted from the included studies.

	Author, Year (Journal)	Study Design	Sample Size	Technique	Treatment Duration	Molar Intrusion (mm)	Overbite Increase (mm)	Type of TAD
**1**	Ogura et al., 2024 [8]	Retrospective non-randomized clinical study	10	Maxillary molar intrusion with micro-implants	7.1 months (intrusion) + 3.1 years (total)	1.6	4.1	Mini-implants
**2**	Al-Falahi et al., 2018 [12]	Non-randomized clinical trial	15	Maxillary molar intrusion with mini-implants	5.1 ± 1.3 months	2.79 ± 0.46	Not reported	Mini-implants
**3**	Akl et al., 2021 [13]	Randomized clinical trial	20	Maxillary molar intrusion with mini-implants and root resorption evaluation	6 months	Not reported	Not reported	Mini-implants
**4**	Akl et al., 2020 [14]	Randomized clinical trial	20	Maxillary molar intrusion with mini-implants (200 g vs. 400 g force)	6 months	2.42 (200 g), 2.26 (400 g)	2.24 (200 g), 3.15 (400 g)	Mini-implants
**5**	Lee et al., 2024 [15]	Cohort study	56	Maxillary molar intrusion with mini-implants vs. orthognathic surgery	5.2 ± 1.3 months	1.7	4.0 (−0.9 mm after 2 years)	Mini-implants
**6**	Marzouk et al., 2015 [16]	Non-randomized clinical trial	13	Maxillary molar intrusion with zygomatic miniplates	9 ± 2.5 months	3.1 ± 0.74	6.55 ± 1.83	Zygomatic miniplates
**7**	Marzouk et al., 2016 [17]	Cohort study	26	Maxillary molar intrusion with zygomatic miniplates	4 years	3.04	6.93	Zygomatic miniplates
**8**	Peres et al., 2023 [18]	Cohort study	53	Maxillary molar intrusion with mini-implants	Variable	2.67	3.6	Mini-implants
**9**	Akan et al., 2020 [19]	Cohort study	19	Maxillary molar intrusion with zygomatic miniplates and acrylic appliance	9.4 ± 0.7 months	2.32	2.48	Zygomatic miniplates
**10**	Lo-Cao et al., 2025 [20]	Cohort study	29	Invisalign vs. intrusion with TADs (Sydney Intrusion Spring)	28.7 ± 3.9 months	2.09–2.98	2.03–2.87	Mini-implants with SIS
**11**	Guo et al., 2024 [21]	Cohort study	36	Maxillary molar intrusion with TADs	Variable	2.25	Variable	Mini-implants
**12**	Kaku et al., 2019 [22]	Case report	1	Molar intrusion with mini-implants and molar extraction	36 months	1	Improved from −6.0 to 1.5 mm	Palatal mini-implants

**Table 3:** ROBINS I (risk of bias judgements in non-randomized studies of interventions).

	1	3	4	5	6	8	9	10	12
**1. Confounding Bias**									
a. Were the inclusion and exclusion criteria clearly described?									
b. Were the participants selected in an appropriate and unbiased manner?									
c. Were relevant confounding variables considered and adjusted for?									
**2. Intervention Classification Bias**									
a. Was the classification of the intervention blinded for participants and outcome assessors?									
b. Are the methods used to classify the intervention clearly described?									
c. Were the classification methods applied consistently across all participants?									
**3. Bias Due to Deviations from Intended Interventions**									
a. Is the intervention protocol clearly described?									
b. Was the intervention applied according to protocol?									
c. Were deviations from the intervention protocol reported appropriately?									
**4. Attrition Bias**									
a. Was there a systematic loss of participants during follow-up?									
b. Was participant loss similar across comparison groups?									
c. Were sensitivity analyses conducted to assess the impact of participant loss?									
**5. Outcome Measurement Bias**									
a. Were objective and valid measures used to assess outcomes?									
b. Were outcomes assessed in a blinded manner for participants and evaluators?									
c. Were outcome measurement results reported completely and appropriately?									
**6. Reporting Bias**									
a. Were the reported outcomes selected appropriately?									
b. Were the results of all pre-specified analyses reported?									
**7. Inappropriate Outcome Reporting Bias**									
a. Were all important consequences of the interventions considered?									
b. Were all relevant outcomes, including adverse events, reported?									
c. Were sensitivity analyses conducted to assess the impact of missing inappropriate outcome data?									

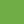
 Yes—Criterion fulfilled; 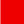 No—Criterion not fulfilled; 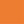 Unclear—Insufficient information to determine; 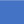 Not applicable—Criterion not relevant to the study.

**Table 4:** RoB 2 (Risk of bias).

	7	11
**1. Bias in the randomization process**		
**a.** Was the generation of the random sequence adequate?		
**b.** Was the allocation adequately concealed until the intervention?		
**c.** Were there any suspicious imbalances in baseline characteristics?		
**2. Bias due to deviations from intended interventions**		
**a.** Were participants and personnel blinded to allocation?		
**b.** Were there important deviations from the protocol?		
**c.** Were the data analyzed according to the original allocation (intention to treat)?		
**3. Bias due to missing outcome data**		
**a.** Were outcome data missing for some participants?		
**b.** Were the missing data related to the outcome?		
**c.** Were appropriate methods used to handle the missing data?		
**4. Bias in outcome measurement**		
**a.** Was the method of measurement appropriate for the outcome?		
**b.** Were outcome assessors blinded to the assigned group?		
**c.** Is it likely that outcome measurement was influenced by knowledge of the intervention?		
**5. Bias in selection of the reported result**		
**a.** Were all pre-specified outcomes reported?		
**b.** Were the reported outcomes selected after knowing the results?		

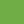
 Yes—Criterion fulfilled.

**Table 5:** JBI (Joanna Briggs Institute).

	2
Were the two groups similar and recruited from the same population?	
Were the exposures measured similarly to assign participants to exposed and unexposed groups?	
Was the exposure measured in a valid and reliable manner?	
Were the conditions that were present at the start of the study clearly identified?	
Were the outcomes of interest identified at the start of the study?	
Were confounding factors identified and adjusted for?	
Were the outcomes measured in a valid and reliable way?	
Was the follow-up period long enough for outcomes to occur?	
Was follow-up complete, and if not, were the reasons for loss to follow-up described?	
Were appropriate methods used for statistical analysis?	
Was the study approved by an ethics committee, and was informed consent obtained from participants?	

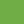
 Yes—Criterion fulfilled; 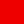 No—Criterion not fulfilled; 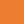 Unclear—Insufficient information to determine; 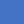 Not applicable—Criterion not relevant to the study.

## Data Availability

No new data were created or analyzed in this study.

## Abbreviations

The following abbreviations are used in this manuscript:

TADs	Dispositivos de anclaje temporal
MAA	Mordida Abierta Anterior
